# Prediction of Past SARS-CoV-2 Infections: A Prospective Cohort Study Among Swiss Schoolchildren

**DOI:** 10.3389/fped.2021.710785

**Published:** 2021-08-16

**Authors:** Jacob Blankenberger, Sarah R. Haile, Milo A. Puhan, Christoph Berger, Thomas Radtke, Susi Kriemler, Agne Ulyte

**Affiliations:** ^1^Epidemiology, Biostatistics and Prevention Institute, University of Zurich, Zurich, Switzerland; ^2^Division of Infectious Diseases and Hospital Epidemiology, University Children Hospital Zurich, Zurich, Switzerland

**Keywords:** SARS-CoV-2, serology, predictors, COVID-19, symptoms, exposure

## Abstract

**Objective:** To assess the predictive value of symptoms, sociodemographic characteristics, and SARS-CoV-2 exposure in household, school, and community setting for SARS-CoV-2 seropositivity in Swiss schoolchildren at two time points in 2020.

**Design:** Serological testing of children in primary and secondary schools (aged 6–13 and 12–16 years, respectively) took place in June–July (T1) and October–November (T2) 2020, as part of the longitudinal, school-based study *Ciao Corona* in the canton of Zurich, Switzerland. Information on sociodemographic characteristics and clinical history was collected with questionnaires to parents; information on school-level SARS-CoV-2 infections was collected with questionnaires to school principals. Community-level cumulative incidence was obtained from official statistics. We used logistic regression to identify individual predictors of seropositivity and assessed the predictive performance of symptom- and exposure-based prediction models.

**Results:** A total of 2,496 children (74 seropositive) at T1 and 2,152 children (109 seropositive) at T2 were included. Except for anosmia (odds ratio 15.4, 95% confidence interval [3.4–70.7]) and headache (2.0 [1.03–3.9]) at T2, none of the individual symptoms were significantly predictive of seropositivity at either time point. Of all the exposure variables, a reported SARS-CoV-2 case in the household was the strongest predictor for seropositivity at T1 (12.4 [5.8–26.7]) and T2 (10.8 [4.5–25.8]). At both time points, area under the receiver operating characteristic curve was greater for exposure-based (T1, 0.69; T2, 0.64) than symptom-based prediction models (T1, 0.59; T2, 0.57).

**Conclusions:** In children, retrospective identification of past SARS-CoV-2 infections based on symptoms is imprecise. SARS-CoV-2 seropositivity is better predicted by factors of SARS-CoV-2 exposure, especially reported SARS-CoV-2 cases in the household. Predicting SARS-CoV-2 seropositivity in children in general is challenging, as few reliable predictors could be identified. For an accurate retrospective identification of SARS-CoV-2 infections in children, serological tests are likely indispensable.

**Trial registration number:** NCT04448717.

## Introduction

Clinical manifestation of severe acute respiratory syndrome coronavirus type 2 (SARS-CoV-2) infections in children is milder and less specific than in adults (1–4). Therefore, identifying children with a SARS-CoV-2 infection is challenging, and many acute infections remain unnoticed (5).

Serological testing is useful to identify past SARS-CoV-2 infections. Its importance to understand the clinical and epidemiological characteristics of the full spectrum of SARS-CoV-2 in children has already been addressed (6). Predictors for SARS-CoV-2 seropositivity could be valuable indicators for identifying past infections in children without the need of serological testing. As such, they could facilitate diagnosis of pediatric multisystem inflammatory syndrome temporally associated with SARS-CoV-2 (PIMS-TS), a disease typically occurring 3–4 weeks after a SARS-CoV-2 infection (7), or long-COVID (8).

In contrast to those in adults (9–12), associations of SARS-CoV-2 seropositivity with history of symptoms in children are less clear (9, 11, 13). Retrospectively assessed symptoms might be less reliable to predict past SARS-CoV-2 infections in children. Evidence on non-clinical factors (e.g., SARS-CoV-2 cases in the environment and sociodemographic characteristics) associated with seropositivity in children is scarce (9, 11, 13) or restricted to specific settings (14, 15). In addition, many of these serology studies were conducted at an early stage of the pandemic and therefore represent only the situation at a specific time.

As part of the longitudinal, school-based, prospective cohort study *Ciao Corona*, approximately 2,500 schoolchildren of primary and secondary schools (aged 6–16 years) in the canton of Zurich, Switzerland, have been tested for SARS-CoV-2 antibodies in June–July and October–November 2020. We aimed to assess the predictive value of both clinical and non-clinical variables on SARS-CoV-2 seropositivity in schoolchildren at the two different time points, representing different epidemiological situations in the course of the SARS-CoV-2 pandemic in Switzerland. In particular, we (1) assessed and compared the individual predictive value of reported symptoms, and factors of SARS-CoV-2 exposure (sociodemographic characteristics, household living conditions, and reported SARS-CoV-2 cases in household, school, and community) and (2) compared a symptom-based vs. exposure-based prediction approach to predict SARS-CoV-2 seropositivity.

## Materials and Methods

**Figure 1:**
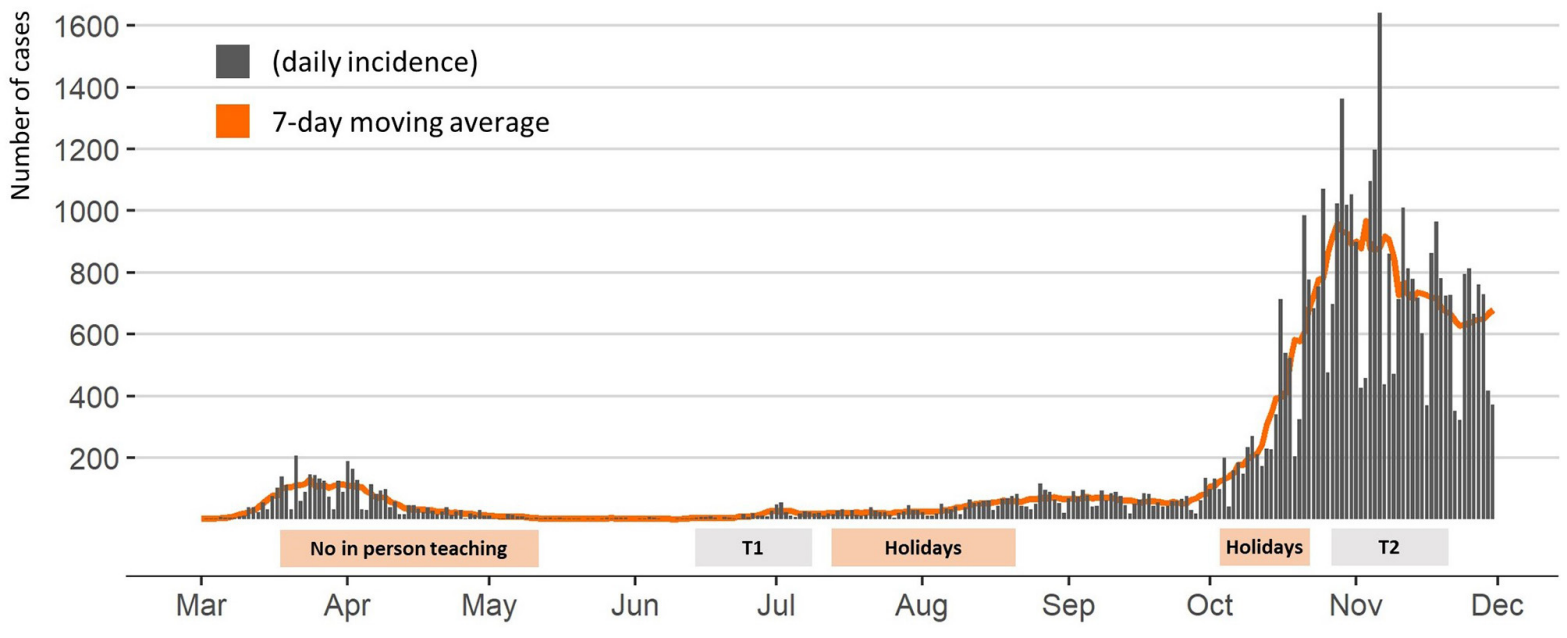
Daily incidence of SARS-CoV-2 cases in the canton of Zurich, Switzerland, and school operation between January and December 2020. T1, June 16 to July 9, 2020; T2, October 26 to November 19, 2020.

The detailed protocol of the *Ciao Corona* study is reported elsewhere (16). *Ciao Corona* is part of *Corona Immunitas*, a nationally coordinated research network in Switzerland (17). The canton of Zurich, where the study is conducted, has a linguistically and ethnically diverse population of 1.5 million, living in both rural and urban areas. The first SARS-CoV-2 epidemic wave peaked at the end of March and was followed by a period of low daily incidence in May–June 2020. Thereafter, infection numbers have steadily increased, resulting in steep growth of new infections starting at the beginning of October 2020. In-person teaching in schools was interrupted only from March 16 until May 10, 2020, along with other containment measures that were gradually lifted at the end of April 2020 and progressively reinstated as the number of cases increased in October 2020. A timeline of SARS-CoV-2 incidence and school operation is given in Figure 1. In-person teaching in schools was interrupted only from March 16 until May 10, 2020. Schools were reopened for June–July 2020 and, afterwards, after summer holiday in August 2020 with implemented preventive measures (e.g., distancing rules in classrooms and teachers' rooms, no mixing of classes, reduction of large group activities, and school-specific contact tracing system). The measures varied, but all schools required children to stay at home if they were ill unless their symptoms were very mild (such as a runny nose or mild cough). As the number of community SARS-CoV-2 cases increased in October 2020, adults in schools were required to wear masks from October 19 and secondary schoolchildren (older than 12 years) from November 2, 2020.

### Study Design and Participants

Prior to the first round of testing, we randomly selected primary schools within the canton of Zurich, stratified by region, as well as the closest geographically matched secondary school, and we invited them to participate in the study. To represent the full range of the Swiss education system, both public and private (about 10%) schools were eligible. After the initial invitation round, overall school participation was assessed, and additional schools were randomly selected within the required regions. From a total of 156 invited schools, 55 agreed to participate, of which two were private schools. Selection of classes was stratified by school level (lower, middle, and upper) and limited to grades 1–2 (typically attended by 6- to 8-year-old children) in the lower school level, grades 4–5 (typically attended by 9- to 11-year-old children) in the middle school level, and grades 7–8 in the upper school level (typically attended by 12- to 14-year-old children). All children of the randomly selected classes were eligible.

### Serological Tests

The outcome measure was the binary (positive/negative) SARS-CoV-2 antibody test result. Venous blood was collected from the children at schools at two time points: between June 16 and July 9, 2020 (T1), and between October 26 and November 19, 2020 (T2). Samples were analyzed using ABCORA 2.0 test (sensitivity 94.3%, specificity 99.0%), a Luminex-based binding assay developed by the Institute of Medical Virology, University of Zurich, measuring the binding of IgG, IgA, and IgM plasma antibodies against subunits of the SARS-CoV-2 S-Protein (S1, S2, and RBD) and nucleoprotein, yielding 12 different measurements. Samples were defined as seropositive for SARS-CoV-2 if at least two of the 12 parameters were above the cutoff value. Test performance was verified on separate validation cohorts of pre-pandemic healthy adults, pre-pandemic children, and individuals with documented SARS-CoV-2 infection (18, 19).

**Figure 2:**
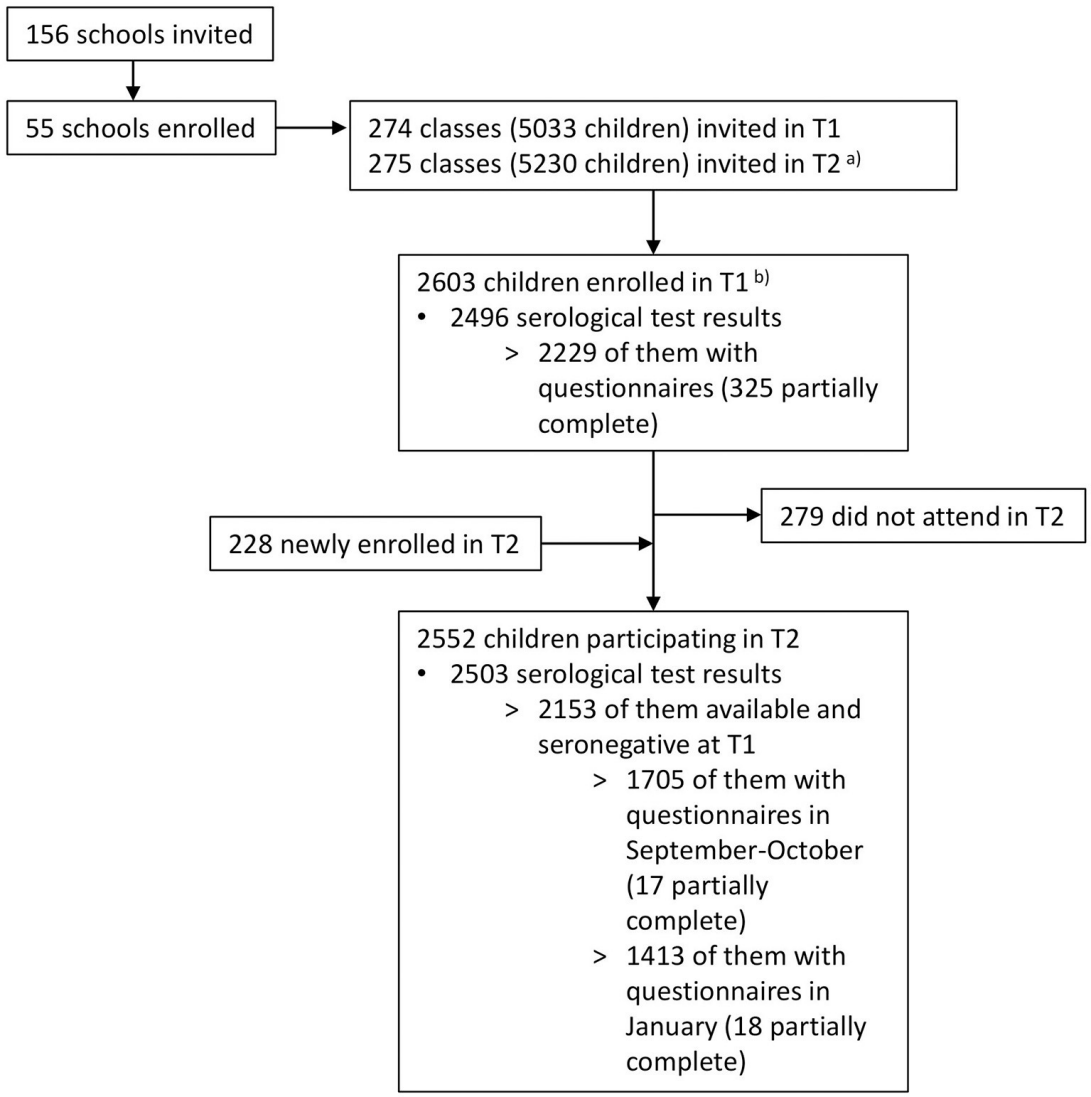
Flowchart of study participants. T1, June 16 to July 9, 2020; T2, October 26 to November 19, 2020. ^a^Some classes were split or rearranged into multiple classes after summer break. ^b^Eighteen of these children were enrolled from late August to early September 2020 (12 serology results, 18 questionnaires).

Figure 2 shows a flowchart of the study participants. Serological results were available for 2,496 children at T1 (2,484 children tested in June–July and additional 12 children in August–September 2020). Of these, 2,153 children seronegative at T1 were also tested in October–November 2020 and included in the T2 analysis.

### Questionnaires and Other Data Sources

Information on sociodemographic characteristics, household size, SARS-CoV-2-compatible symptoms, and SARS-CoV-2 infections confirmed by reverse transcriptase polymerase chain reaction (RT-PCR) of participants and their household members was collected in baseline and follow-up questionnaires. Parents were asked to fill in baseline questionnaires in parallel to T1, covering SARS-CoV-2-compatible symptoms and RT-PCR-confirmed infections since January 2020 (complete for 1,904, partially complete for 325 participants). Data on symptoms and positive SARS-CoV-2 tests following T1 were obtained from follow-up questionnaires in September–October 2020 (complete for 1,688, partially complete for 17 participants) and if available in January 2021 (complete for 1,413, partially complete 18 participants). Symptoms and positive SARS-CoV-2 tests reported to have occurred at least 2 weeks prior to antibody testing at T2 were included, allowing time for potential seroconversion (20).

Information on RT-PCR-confirmed SARS-CoV-2 cases in schools was reported by school principals at the start of each round of testing (all 55 schools at T1 and 53 schools at T2).

Cumulative incidence of RT-PCR-confirmed cases of all age groups 14 days before the testing dates was obtained on the postal code level of children's home address from the health directorate of the canton of Zurich.

### Statistical Analysis

Categorical variables are presented as count and percentage, and continuous variables as mean with standard deviation, stratified by seropositivity.

**Figure 3:**
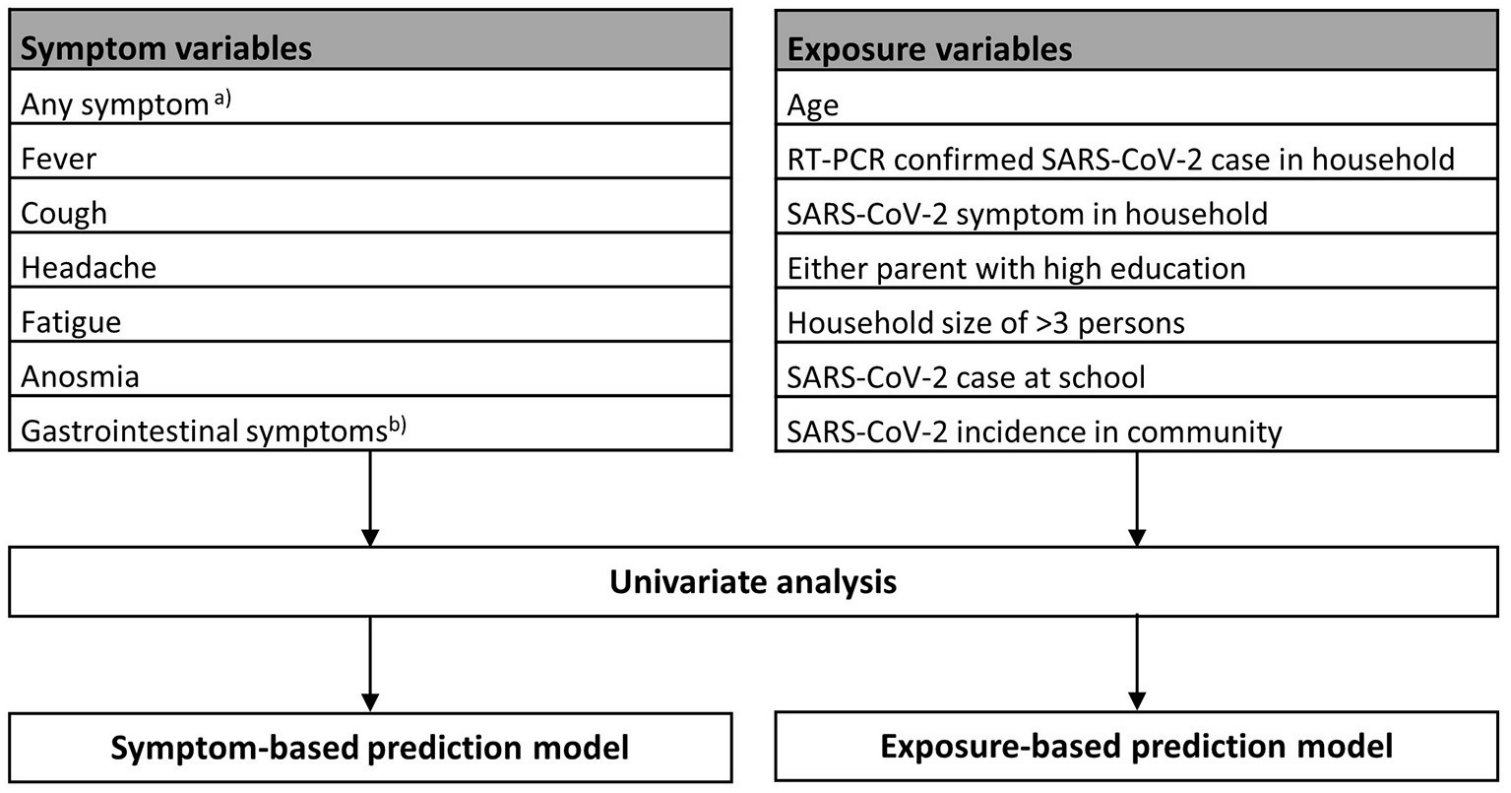
Univariate and multivariable logistic regression analyses of symptom and exposure variables at T1 and T2. ^a^Any of the following symptoms: fever, cough, runny nose, sneezing, sore throat, shortness of breath, headache, myalgia, fatigue, loss of appetite, nausea, emesis, diarrhea, upset stomach, and anosmia. ^b^Any of the following symptoms: loss of appetite, nausea, emesis, diarrhea, and upset stomach.

Logistic regression was used to assess the predictive effect for seropositivity of a selected subset of variables. The selection of symptom variables was based on most typical SARS-CoV-2 related symptoms in children reported in literature (4, 9, 13). Exposure variables were chosen to represent potential exposure to SARS-CoV-2 due to RT-PCR-confirmed cases in the household, school, and community; household size (21); socioeconomic status (22) (approximated by education of parents); and behavior and contact patterns depending on the child's age (23). Included variables are listed in Figure 3. Apart from age (in years) and rate of SARS-CoV-2 cases in the community (cumulative incidence of RT-PCR-confirmed SARS-CoV-2 cases per 1,000 inhabitants), included variables were binary. Analysis was performed for serology results at T1 (representing January–June/July 2020) and T2 (representing August–October 2020) separately, to assess possible differences in the variable's predictive value depending on the epidemiological situation.

Association of each individual variable with SARS-CoV-2 seropositivity was assessed using univariate logistic regression. To assess and compare total predictive value per category (total predictive value of all symptom variables vs. all exposure variables) for each T1 and T2, two separate multivariable logistic regression models were built. In both univariate and multivariable analyses, only cases with complete data on the respective variables were analyzed (symptom- and exposure-based models at T1 2,223 and 2,117 cases, and symptom- and exposure-based models at T2 1,768 and 1,593 cases, respectively). Overall predictive performance of the models was assessed using the Brier score, a proper score assessing the difference of the predicted probability with the actual binary outcome (Brier score = mean (observed – predicted)^2^) (24). Discrimination, the ability of the prediction model to distinguish between cases with and without the outcome, was assessed using area under the receiver operating characteristic (ROC) curve (AUC) (24).

To assess the models' practical usefulness for identifying children with past SARS-CoV-2 infections, we also calculated positive predictive value (PPV) and negative predictive value (NPV) for all categorical variables with statistically significant association in univariate analyses. Furthermore, we used the highest Youden's J to determine optimal thresholds for the multivariable models (25), and we calculated the PPV and NPV for SARS-CoV-2 seropositivity based on these thresholds.

Specific SARS-CoV-2-compatible symptoms in household members were assessed for T1 only. These variables were analyzed in univariate analysis and not included in the multivariable models, to ensure the model's comparability between T1 and T2. Of the reported symptoms in household members, we selected fever, cough, anosmia, fatigue, and myalgia to be assessed for their association with seropositivity in children at T1, as these symptoms are most typically associated with a SARS-CoV-2 infection in adults (9, 12) (75% of household members reporting symptoms were older than 18 years).

## Results

Of the 2,496 participants tested for SARS-CoV-2 antibodies at T1, 74 (3.0%) were seropositive. Of the 2,153 seronegative children at T1 with an available test result at T2, 109 (5.1%) were seropositive. The detailed seroprevalence results of both testing rounds are reported elsewhere (5, 26).

Reported symptoms, sociodemographic characteristics, and presence of factors of SARS-CoV-2 exposure in household, school, and community are shown in Table 1. Six of 55 schools reported at least one RT-PCR-confirmed case among their pupils or school personnel (one to six cases per school) between January and June–July and 21/53 schools from August to October (one to seven cases per school). Children resided in 101 different post-code areas (1–112 children per post-code area) for which cumulative incidence per 1,000 inhabitants was 0.0–6.1 at T1 and 0.0–24.6 at T2.

**Table 1:** Demographics, symptoms, and exposure information in seronegative and seropositive children.

	**T1**			**T2**		
**Variable**	**Complete**	**Seronegative**	**Seropositive**	**Complete**	**Seronegative**	**Seropositive**
SARS-CoV-2 serology	2,496	2,422 (97)	74 (3.0)	2,153	2,044 (95)	109 (5.1)
**Demographic characteristics**						
Age^a^	2,496	12.0 (±2.5)	10.1 (±2.5)	2,153	11.3 (±2.5)	11.4 (±2.3)
Female sex	2,496	1,246 (51)	34 (46)	2,153	1,057 (52)	55 (51)
**RT-PCR diagnosis and reported symptoms in child**						
Positive PCR test	2,223	0 (0.0)	1 (1.4)	1,768	0 (0.0)	2 (1.8)
Any symptoms^b^	2,223	1569 (73)	51 (74)	1,768	311 (19)	20 (24)
Fever	2,223	558 (26)	22 (32)	1,768	80 (4.8)	6 (7.1)
Cough	2,223	805 (37)	27 (39)	1,768	130 (7.7)	8 (9.4)
Runny nose/sneezing	2,223	963 (45)	35 (51)	1,768	225 (13)	9 (11)
Sore throat	2,223	737 (34)	21 (30)	1,768	163 (9.7)	9 (11)
Shortness of breath	2,223	63 (2.9)	1 (1.4)	1,768	8 (0.5)	0 (0.0)
Headache	2,223	821 (38)	23 (33)	1,768	113 (6.7)	7 (8.2)
Myalgia	2,223	292 (14)	7 (10)	1,768	26 (1.5)	2 (2.4)
Fatigue	2,223	493 (23)	19 (28)	1,768	80 (4.8)	4 (4.7)
Gastrointestinal symptoms^c^	2,223	541 (25)	13 (19)	1,768	83 (4.9)	5 (5.9)
Anosmia	2,223	33 (1.5)	0 (0.0)	1,768	4 (0.2)	3 (3.5)
**Socioeconomic characteristics of the household of child**						
Either parent with high education	2,124	1,469 (71)	46 (72)	1,859	1,323 (74)	58 (67)
Either parent Swiss	1,961	1,737 (92)	57 (91)	1,710	1,507 (74)	70 (65)
Household size >3 persons	2,227	1,811 (84)	53 (77)	1,942	1,389 (84)	70 (85)
RT-PCR diagnosis and reported symptoms in household members^*^						
RT-PCR-confirmed SARS-CoV-2 case in household	2,227	29 (1.3)	10 (15)	1,772	21 (1.2)	10 (12)
SARS-CoV-2 symptom in household	2,227	1,596 (74)	61 (88)	1,766	328 (20)	16 (21)
Fever	2,227	702 (33)	32 (46)			
Cough	2,227	979 (45)	34 (49)			
Runny nose/sneezing	2,227	993 (46)	32 (46)			
Sore throat	2,227	987 (46)	38 (55)			
Shortness of breath	2,227	150 (7.0)	15 (22)			
Headache	2,227	949 (44)	39 (57)			
Myalgia	2,227	433 (20)	28 (41)			
Fatigue	2,227	664 (31)	32 (46)			
Gastrointestinal symptoms	2,227	568 (26)	27 (39)			
Anosmia	2,227	79 (3.7)	17 (25)			
**SARS-CoV-2 in school**						
<1 SARS-CoV-2 case at school	2,496	257 (11)	14 (19)	2,059	755 (39)	35 (33)
**SARS-CoV-2 in community**						
SARS-CoV-2 incidence at community^d^	2,224	2.4 (1.0)	2.66 (0.9)	1,940	10.8 (5.0)	11.9 (4.6)

*If not indicated otherwise, data are presented as count (%)*.

*T1, January–June/July 2020; T2, August–October/November 2020*.

* *Results of individual symptoms in household were not available for T2*.

a *Presented as mean (±standard deviation)*.

b *Any of the following symptoms: fever, cough, runny nose, sneezing, sore throat, shortness of breath, headache, myalgia, fatigue, loss of appetite, nausea, emesis, diarrhea, upset stomach, and anosmia*.

c *Any of the following symptoms: loss of appetite, nausea, emesis, diarrhea, and upset stomach*.

d *Cumulative incidence of SARS-CoV-2 cases on community level per 1,000 inhabitants, presented as median (interquartile range)*.

### Univariate Prediction Analysis

Results of the univariate analysis of symptoms and exposure variables at T1 and T2 are shown in Table 2.

**Table 2:** Univariate analysis of symptom and exposure variables assessing the predictive effect on SARS-CoV-2 seropositivity.

	**T1**	**T2**
**Variable**	**Odds ratio (95% CI)**	**Odds ratio (95% CI)**
**Symptom variables**		
Any symptom^a^	1.06 (0.61–1.8)	**1.7 (1.03–2.7)**
Fever	1.3 (0.80–2.2)	1.8 (0.79–4.0)
Cough	1.08 (0.66–1.8)	1.7 (0.88–3.3)
Headache	0.81 (0.49–1.3)	**2.0 (1.03–3.9)**
Fatigue	1.3 (0.75–2.2)	1.4 (0.61–3.4)
Anosmia	–^d^	**15.4 (3.4–70.7)**
Gastrointestinal symptoms^b^	0.69 (0.38–1.3)	1.4 (0.58–3.2)
**Exposure variables**		
Age	**0.87 (0.79–0.96)**	1.01 (0.92–1.2)
RT-PCR-confirmed SARS-CoV-2 case in household	**12.4 (5.8–26.7)**	**10.8 (4.5–25.8)**
SARS-CoV-2 symptom in household	**2.7 (1.3–5.6)**	1.04 (0.58–1.9)
Either parent with high education	1.03 (0.59–1.8)	0.69 (0.41–1.2)
Household size of >3 persons	0.63 (0.35–1.1)	1.5 (0.70–3.1)
SARS-CoV-2 case at school	**1.9 (1.08–3.6)**	0.58 (0.34–1.00)
SARS-CoV-2 incidence at community^c^	1.2 (0.98–1.5)	1.02 (0.97–1.06)

*Data are presented as odds ratios with 95% confidence interval (CI)*.

*T1, January–June/July 2020; T2, August–October/November 2020*.

*Estimates in bold are statistically significant (p < 0.05)*.

a *Any of the following symptoms: fever, cough, runny nose, sneezing, sore throat, shortness of breath, headache, myalgia, fatigue, loss of appetite, nausea, emesis, diarrhea, upset stomach, and anosmia*.

b *Any of the following symptoms: loss of appetite, nausea, emesis, diarrhea, and upset stomach*.

c *Cumulative incidence of SARS-CoV-2 cases on community level per 1,000 inhabitants*.

d *Estimate is 0.00000072 (95% CI 0–infinity), as no cases of anosmia were reported among seropositive children at T1*.

Analysis of symptoms showed no statistically significant predictive effect for any of the variables at T1. Of three variables at T2 for which a statistically significant predictive effect was observed, odds ratios (ORs) were the highest for anosmia, followed by headache, and the presence of any SARS-CoV-2-compatible symptoms (fever, cough, rhinorrhea, sore throat, shortness of breath, headache, myalgia, fatigue, loss of appetite, nausea, emesis, diarrhea, upset stomach, and anosmia). At T2, PPVs for anosmia, presence of any symptoms, and headache were 42.9, 7.0, and 8.6%, respectively; and NPVs were 95.3, 95.7, and 95.5%, respectively. Given the seroprevalence of 5.1% at T2 in our cohort, neither of these variables adds significant information to exclude past SARS-CoV-2 infections.

Among exposure variables, an RT-PCR-confirmed SARS-CoV-2 case of a household member had the time strongest predictive effect on seropositivity in both T1 and T2. At T1, a weaker but statistically significant predictive effect was further observed for SARS-CoV-2-compatible symptoms in household members, followed by reported SARS-CoV-2 cases at school and age (lower odds of SARS-CoV-2 seropositivity with older age). RT-PCR-confirmed SARS-CoV-2 cases in household members showed also a comparatively high PPV in both T1 (21.6%) and T2 (30.8%), in contrast to symptoms in household members (PPV: 3.6%) and reported SARS-CoV-2 cases at school (PPV: 5.7%) at T1. SARS-CoV-2 infections at T1 were most reliably ruled out when household members were asymptomatic (NPV: 98.6%), followed by the absence of SARS-CoV-2 cases in school (NPV: 97.3%) and SARS-CoV-2 cases at home (NPV: 97.3%).

### Multivariable Prediction Models

Coefficients of variables of the symptom- and exposure-based prediction models are displayed in Table 3.

**Table 3:** Multivariable logistic regression models assessing the predictive effect of symptoms and of exposure variables on SARS-CoV-2 seropositivity.

	**T1**	**T2**
**Variable**	**Odds ratio (95% CI)**	**Odds ratio (95% CI)**
**Symptom-based prediction model**		
(Intercept)	**0.03 (0.02–0.05)**	**0.04 (0.03–0.06)**
Any symptom^a^	1.1 (0.55–2.2)	1.4 (0.67–3.1)
Fever	1.5 (0.82–2.8)	1.03 (0.37–2.9)
Cough	0.98 (0.54–1.8)	1.1 (0.47–2.6)
Headache	0.71 (0.39–1.3)	1.5 (0.59–3.9)
Fatigue	1.6 (0.84–2.9)	0.69 (0.23–2.1)
Anosmia	–^c^	**12.2 (2.3–64.0)**
Gastrointestinal symptoms^b^	0.6 (0.3–1.2)	0.69 (0.24–2.0)
**Exposure-based prediction model**		
(Intercept)	**0.11 (0.02–0.62)**	**0.04 (0.01–0.17)**
Age	**0.87 (0.78–0.96)**	1.01 (0.91–1.1)
RT-PCR-confirmed SARS-CoV-2 case in household	**8.0 (3.4–19.0)**	**10.1 (4.2–24.6)**
SARS-CoV-2 symptom in household	**2.5 (1.1–5.5)**	0.97 (0.53–1.7)
Either parent with high education	0.80 (0.45–1.4)	0.62 (0.36–1.06)
Household size of >3 persons	**0.52 (0.29–0.95)**	1.4 (0.66–3.0)
SARS-CoV-2 case at school	**2.6 (1.3–5.3)**	0.59 (0.34–1.02)
SARS-CoV-2 incidence at community^d^	0.97 (0.74–1.3)	1.02 (0.98–1.07)

*Data are presented as odds ratio with 95% confidence interval (CI)*.

*Estimates in bold are statistically significant (p < 0.05)*.

a *Any of the following symptoms: fever, cough, runny nose, sneezing, sore throat, shortness of breath, headache, myalgia, fatigue, loss of appetite, nausea, emesis, diarrhea, upset stomach, and anosmia*.

b *Any of the following symptoms: loss of appetite, nausea, emesis, diarrhea, and upset stomach*.

c *Estimate is 0.00000066 (95% CI 0–infinity), as no cases of anosmia were reported among seropositive children at T1*.

d *Cumulative incidence of SARS-CoV-2 cases on community level per 1,000 inhabitants*.

The Brier score was slightly lower for the exposure-based compared model with the symptom-based prediction model at both T1 (symptom-based model: 0.030, exposure-based model: 0.028) and T2 (symptom-based model: 0.045, exposure-based model 0.041), indicating a better overall prediction performance of the exposure-based model.

**Figure 4:**
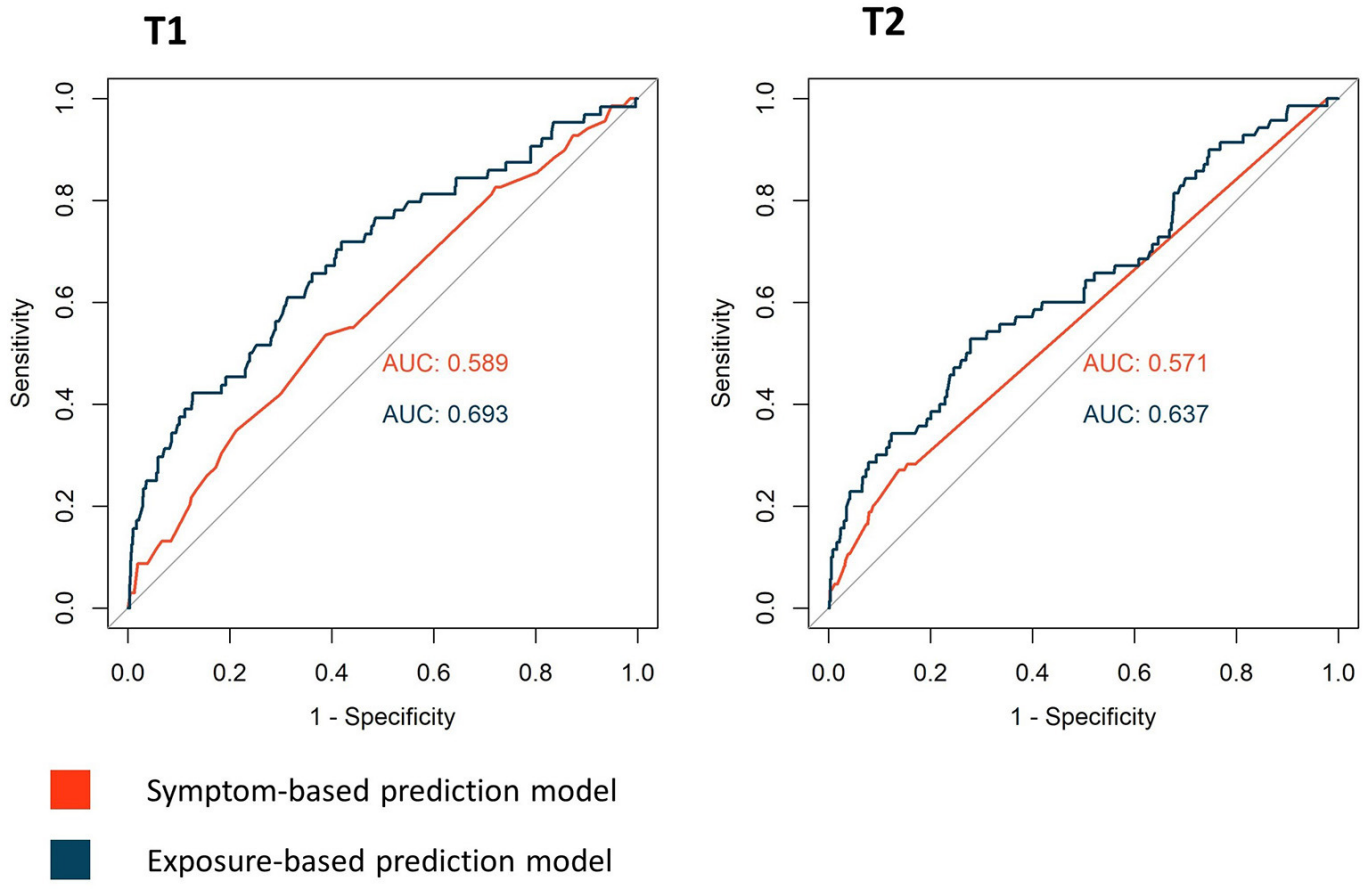
Receiver operating characteristics (ROC) curves for the symptom- and exposure-based prediction models for T1 and T2.

Discriminative abilities of the models are illustrated with ROC curves in Figure 4; optimal thresholds and resulting PPV and NPV are in Table 4. Exposure-based predictions showed superior discriminative ability than the symptom-based models, with an AUC of 0.59 [95% confidence interval (CI) 0.52–0.66] for the symptom-based model and 0.69 (95% CI 0.62–0.76) for the exposure-based model at T1; and 0.57 (95% CI 0.52–0.62) for the symptom-based model and 0.64 (95% CI 0.56–0.70) for the exposure-based model at T2.

**Table 4:** Positive and negative predictive values of the symptom- and exposure-based prediction models based on optimal threshold determined by the highest Youden's J.

**Model**	**Threshold^a^**	**PPV (%)**	**NPV (%)**
**T1**			
Symptom-based prediction model	≥0.0319	4.2%	97.6%
Exposure-based prediction model	≥0.0257	5.1%	98.5%
**T2**			
Symptom-based prediction model	≥0.0553	9.1%	95.9%
Exposure-based prediction model	≥0.0431	8.0%	97.1%

*T1, January–June/July 2020; T2, August–October/November 2020; PPV, positive predictive value; NPV, negative predictive value*.

a *Threshold for the predicted probability of SARS-CoV-2 seropositivity*.

### Predictions Based on Symptoms in Household

In univariate analysis of symptoms of household members, the predictive effect on the child's seropositivity at T1 was statistically significant for all assessed symptoms except cough. The predictive effect was the highest for anosmia (OR 8.6, 95% CI 4.7–15.5), followed by myalgia (OR 2.7, 95% CI 1.7–4.4), fatigue (OR 1.9, 95% CI 1.2–3.2), and fever (OR 1.8, 95% CI 1.1–2.9).

## Discussion

In this population-based study, we described the predictive effect of individual symptoms and factors of SARS-CoV-2 exposure for SARS-CoV-2 seropositivity in schoolchildren. We identified RT-PCR-confirmed infections of household members to be the most consistent predictor for SARS-CoV-2 seropositivity in children and showed that risk factors for SARS-CoV-2 exposure predicted seropositivity in children better than parent-reported history of symptoms. These findings can provide some help in identifying past SARS-CoV-2 infections in children and form the foundation for future attempts to build prediction models for SARS-CoV-2 seropositivity in children.

Predictive value of symptoms for SARS-CoV-2 seropositivity was low in our study. Coefficients varied greatly between T1 and T2 analyses; and in line with observations by Tönshoff et al. (11), none of the assessed symptoms were significantly associated with SARS-Co-2 seropositivity in children at T1. T2 results (see Table 2: any symptom, anosmia, and headache) and other studies (9, 13) show that, in some contexts, significant associations of some symptoms with seropositivity can be identified. However, based on PPV, with the exception of anosmia, presence of any or any specific symptoms was an unreliable indicator of a past SARS-CoV-2 infection in our cohort. The longer the time periods for symptom recall, the less precise they can be expected to be for the identification of past SARS-CoV-2 infections, as the likelihood of children experiencing symptoms not related to SARS-CoV-2 increases. Discriminative ability of the symptom-based models was low, and even with the optimal threshold for classification, a substantial number of cases would be misclassified. For an identification of past infections over a period of several months (i.e., at T1 and T2 symptoms were assessed over 6–7 and 3–4 months, respectively), symptom-based prediction approaches appear too unreliable.

The predictive performance of the exposure-based models was generally better than that of the symptom-based models. In line with other studies (27, 28), our results indicate that information on SARS-CoV-2 situation in the household can be valuable for identifying SARS-CoV-2 infections in children. In particular, RT-PCR-confirmed SARS-CoV-2 cases in household members proved to be consistent predictors for seropositivity in children, with similar effect sizes in T1 and T2, and also in comparison with other studies (13, 29). In contrast to the child's symptoms, most individual symptoms in household members were significantly associated with the child's seropositivity, underlining the importance of collecting this information if SARS-CoV-2 infections in household members are not laboratory confirmed.

A few other observations regarding our assessment of exposure variables as predictors for SARS-CoV-2 seropositivity are worth mentioning. Attending a school, with at least one reported SARS-CoV-2 case, was significantly associated with seropositivity at T1, but not at T2. The extent of school-related SARS-CoV-2 transmission is not yet fully understood; however, current evidence suggests that in-person teaching does not play a particularly strong role in amplifying transmission (5, 30). While some causal effects of SARS-CoV-2 cases at school cannot be ruled out, PPV and NPV as well as our observations from T2 (when schools were open and 21/53 (38%) schools reported at least one RT-PCR-confirmed SARS-CoV-2 case) suggest that this variable is likely of little relevance for reliable predictions of SARS-CoV-2 seropositivity. We further found no significant association of SARS-CoV-2 seropositivity and SARS-CoV-2 cases at the community level, although average cumulative incidence on community level was higher in seropositive compared with seronegative children at both T1 and T2. Similar effects were observed for socioeconomic status, which was not significantly associated with seropositivity at both time points. While likely being of too little relevance for prediction, some socioeconomic gradients (see Table 1: foreign origin and education of parents at T2) in distribution of SARS-CoV-2 cases as also documented elsewhere (31) in children cannot be ruled out.

In general, predicting SARS-CoV-2 seropositivity in children is challenging, which is reflected by the lack of reliable individual predictors and low accuracy of the multivariable models developed in this study. Accurate serological tests will remain indispensable for an accurate retrospective identification of SARS-CoV-2 infections in children. The predictive value of both symptoms and exposure variables can change over time. Specificity of symptoms for prediction of SARS-CoV-2 seropositivity depends on the prevalence of symptoms unrelated to SARS-CoV-2, which can change seasonally (e.g., flu season in winter and allergy season in spring). The predictive effect of the exposure variables on the other hand is likely dependent on the epidemiological situation and implemented protective measures. For instance, as lockdown measures in Switzerland were in place in March–April 2020, household transmission might have been more common before T1 compared with T2 (32). This could to some extent explain the significant association of seropositivity with household symptoms at T1, which was absent at T2. Finally, transmission dynamics are changing in many countries due to progressing vaccination. With an increasing number of adults with partial or complete immunity, SARS-CoV-2 infections in children might be linked to infections in household members less frequently, and a higher proportion of infections might take other routes of transmission.

This study has some limitations. Due to the low number of seropositive children in both testing rounds, the power of the statistical models is limited and allowed the assessment of only several predictive variables. Although the antibody test used in our study has shown superior performance measures in comparison with other SARS-CoV-2 antibody tests (18), some misclassifications are possible. Misclassification was likely higher at T1 (PPV of the test 74.5%, NPV 99.8%) compared with T2 (PPV 83.5%, NPV 99.7%), due to lower seroprevalence at T1. False-negative results could have predominantly affected children with asymptomatic and mild infection, as loss of antibodies can occur at an early stage of convalescence (33). Furthermore, as questionnaires asked for symptoms retrospectively over a time of several months, recall bias is likely. Symptoms of children were retrospectively reported by parents, which could have been different to what the children perceived themselves (34) and is also dependent on how much attention was given to symptoms in general. All those factors could increase the noise and alter effect sizes.

## Conclusion

In children, the identification of past SARS-CoV-2 infections based on retrospectively reported symptoms and exposure factors is generally imprecise. Factors of SARS-CoV-2 exposure, especially history of confirmed SARS-CoV-2 infections in the household, predict seropositivity in children better than the child's symptoms. Typical SARS-CoV-2 symptoms in adult household members could to some extent be a predictor for a child's SARS-CoV-2 infection if RT-PCR diagnosis information is not available. For an accurate retrospective identification of SARS-CoV-2 infections in children, serological tests remain indispensable.

## Data Availability Statement

The datasets presented in this article are not readily available. Raw data supporting the conclusions of this article will be made available by the authors, on reasonable request. Requests to access the datasets should be directed to Thomas Radtke, thomas.radtke@uzh.ch.

## Ethics Statement

The study involving human participants was reviewed and approved by Ethics Committee of the Canton of Zurich, Switzerland (2020-01336). Written informed consent to participate in this study was provided by the participants' legal guardian.

## Author Contributions

SK and MP initiated the project and preliminary design. SK, MP, CB, TR, JB, and AU developed the design and methodology. SK, AU, TR, and JB recruited study participants, collected, and managed the data. SH and JB developed the statistical analysis plan. JB wrote the first draft of the manuscript. All authors contributed to the design of the study and interpretation of its results, revised, and approved the manuscript for intellectual content.

## Conflict of Interest

The authors declare that the research was conducted in the absence of any commercial or financial relationships that could be construed as a potential conflict of interest.

## Publisher's Note

All claims expressed in this article are solely those of the authors and do not necessarily represent those of their affiliated organizations, or those of the publisher, the editors and the reviewers. Any product that may be evaluated in this article, or claim that may be made by its manufacturer, is not guaranteed or endorsed by the publisher.
